# Process Development of a Model Solvate for Drying Research

**DOI:** 10.1021/acs.oprd.5c00095

**Published:** 2025-08-26

**Authors:** Nicholas H. McCarthy, Norah S. Alsaiari, Thomas Brown, Faiz M. Mahdi, Andrew E. Bayly, Sadie Finn, Frans L. Muller

**Affiliations:** † School of Chemical and Process Engineering, 4468 University of Leeds, Leeds LS2 9JT, U.K.; ‡ Department of Chemistry, College of Science, 112893 Princess Nourah Bint Abdulrahman University, Riyadh 11671, Saudi Arabia; § Bragg Centre for Materials Research, University of Leeds, Leeds LS2 9JT, U.K.; ∥ Pharmaceutical Technology and Development, 468087 AstraZeneca, Hulley Road, Macclesfield SK11 2NA, U.K.

**Keywords:** drying, crystallization, lollipop, solvate, desolvation

## Abstract

Drying of organic solvates remains hard to scale down and fully understand, as (a) residual solvent is typically hard to remove due to high solid-phase transport resistances, and (b) precise control over crystal properties is challenging. These issues are especially relevant to the pharmaceutical sector and its stringent quality criteria. We have identified a Schiff base forming a methanol solvate (derived from *o*-vanillin and *para*-aminobenzoic acid) with chemical complexity representative of pharmaceutical active ingredients that is cost-effective and a straightforward process for its manufacture has been developed. Initial attempts to crystallize the compound resulted in the formation of a slurry with a high yield stress caused by extremely high product supersaturation. By seeding the crystallization and controlling the addition rate of the catalyzing reagent, the process was successfully scaled up into one which was both high-yielding (93% at 1 L scale) and concentrated. These changes altered the crystal morphology, with crystal growth being favored over nucleation, resulting in larger, higher aspect ratio crystals (from 8 to 20). Powder X-ray diffraction (XRD) showed that the solvated Schiff base gradually transformed into a distinct desolvated polymorph, and a quantitative method for assessing solvent content with XRD was developed. The compound is a promising candidate as a model solvate system for drying trials on account of its (a) high-aspect-ratio morphology typical of many organic products; (b) stability at room temperature, facilitating handling and analysis; (c) desolvation temperature exceeding the boiling point of methanol, separating the drying of the free solvent from the period of desolvation. In conclusion, this relatively unexplored Schiff-base was identified as a promising model solvate for studying drying under industrially relevant conditions.

## Graphical abstract

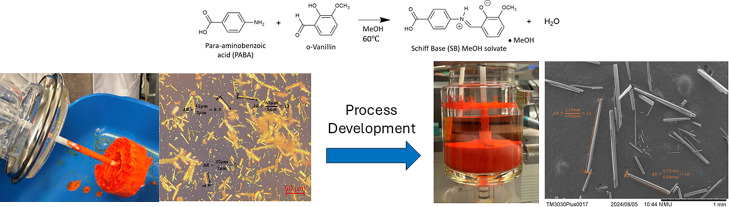

## Introduction

Solvates are a far from negligible element of the solid-state landscape of an active pharmaceutical compound (API), with roughly 10% of organic compounds in the Cambridge Structural Database (CSD) forming a solvate (with an organic solvent).^1^ Furthermore, the increased propensity for solvate formation seen among new drug entities is related to their increasing molecular complexity.^2^ Solvates are structures in which a solvent is incorporated directly into the lattice of a host compound and are usually formed with the solvent of crystallization.^3^ The terms solvatomorph or pseudopolymorph are sometimes used to emphasize that these structures are not true polymorphs (different elemental composition).^4^

The effect of solvent inclusion on the host system can be (a) small, with minor anisotropic expansion to accommodate the solvent molecules, or (b) large, with the inclusion drastically altering the supramolecular packing of the host and forming an entirely new crystal structure.^5^ Solvent in the former tends to be mobile over many crystallographic sites in channels/planes (nonstoichiometric) and gives rise to a continuously variable equilibrium composition over a range of solvent activity. Furthermore, solvent tends to be removable without collapse of the structure (forming an isomorphic desolvate).^6^ Conversely, in the latter, solvent occupies definite positions in the crystal lattice (isolated sites/channels/planes) and is essential for stabilizing the molecular network (stoichiometric). Also, solvent removal almost always leads to lattice disintegration and the formation of a new lattice structure. In practice, the border between the two solvate types is not always sharp, with some systems comprising both traits.^7^ Regardless, solvent inclusion inevitably alters the physicochemical properties of a compound (e.g., solubility, dissolution rate, bioavailability, stability, powder properties^8^). As a result, discovery of solvate forms can prove unwanted, causing significant challenges in the isolation of API’s and key intermediates,^9^ or it can provide opportunities to enhance an API’s physical attributes and/or improve purification and isolation processes.^8^ Overall, this highlights the importance of diligent solid-state screens (of APIs and intermediates) and investigating the equilibrium, structural, and kinetic aspects of solvates and their solvation/desolvation behavior as it can directly impact processing and formulation operations, with the potential to cause scale-up issues if missed during early process development.

Solvates themselves are rarely selected as API candidates due to toxicity/regulatory concerns, although there are several marketed exceptions.^10–17^ Despite this, solvates have several important applications in pharmaceutical processing: (1) improved recovery/purification by the property of solvate formation;^18,19^ (2) facilitate the performance of a unit operation by having a particular crystal habit (e.g., filtration^8^); (3) particle size control, as desolvation can produce crystals with a very homogeneous particle size distribution;^20^ (4) serve as precursors to polymorphs obtained via desolvation.^21–26^ The latter has significance in drying with the unit operation providing a means of accessing crystal forms via the solid state that are inaccessible or overly complex to obtain from direct solution crystallization.^8^

Drying itself is a much-overlooked isolation step within pharmaceutical manufacturing; however, its importance cannot be understated as it can greatly influence the product quality of an API, typically defined by critical quality attributes (CQAs). The process is inherently complex on account of the simultaneous heat, mass, and momentum transfer processes occurring, which are highly dependent on the product to be dried.^27^ Many issues in meeting product specification are only discovered late in development cycles (at larger scale) or when the process is transferred to a different equipment train.^28^ Drying itself can have a direct impact on meeting CQA’s related to particle size, solvent content, and crystal form. The latter two are very important when the material to be dried is a solvate, as (a) there is often difficulty in removing the final traces of potentially toxic organic solvent from the structure due to high resistances to solvent transport in the solid phase (leading to long/unpredictable drying times); (b) if the solvent is more readily removed from the crystal lattice, it can be challenging to control or prevent desolvation due to the inhomogeneity seen in particle beds at scale; and (c) desolvation processes can induce transitions to different, potentially unstable crystal forms. Furthermore, by varying the processing conditions, one can alter the desolvation pathway to produce crystals of a specific form, size, morphology, and defect concentration.^25^ Hence, understanding these kinetic rate processes and structural changes is important, particularly at the gram and kilogram scales, where little research has been conducted.

Of the available solvate drying research, the compounds (other than the solvent) are not usually disclosed.^29–32^ Hydrate drying is a more populated research area, consisting of some known compounds,^33–36^ the classic well-studied example being theophylline,^37–41^ which is cheap, easy to manufacture, and safe. There are many potential model solvate compounds in the literature; however, these tend to suffer from one or more of the following drawbacks: overly complex (tend to be large and expensive), low-yielding crystallizations, and safety concerns. Some of the most promising candidates were niclosamide^42^ and its methanol and acetonitrile solvates (but the process is very dilute) and griseofulvin^43^ and its acetonitrile solvate (but is a carcinogen). Therefore, a clear absence of model solvate compounds exists in pharmaceutical drying literature.

A search for model solvates on the Cambridge Structural Database (CSD) resulted in a number of candidates, including a Schiff base first reported by Kamaal et al.^44^ and later by Tahir et al.^45^ Schiff bases are a group of organic chromophores synthesized by the condensation of a primary amine and an aldehyde/ketone to generate an imine functionality. They have potential applications across many sectors including pharmaceuticals (useful as intermediates and possess multiple bioactive properties, e.g., anticancer, antibacterial), nonlinear optics, analytical chemistry, energy storage, nanomaterials, and catalysis.^46^ The compound, 4-[(3-methoxy-2-oxido-benzylidene)­azaniumyl]­benzoic acid methanol monosolvate (referred to as SB) is formed by reacting PABA (4-aminobenzoic acid) and *o*-vanillin (2-hydroxy-3-methoxybenzaldehyde) in methanol, as shown in Scheme 1.

**Scheme 1:**
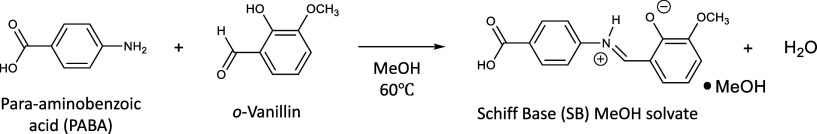
Synthesis of 4-[(3-Methoxy-2-oxido-benzylidene)­azaniumyl] Benzoic Acid Methanol Monosolvate (Schiff Base Solvate, SB·MeOH) from PABA and *o*-Vanillin

The *ortho* hydroxyl group (from vanillin) enables the SB to form an intramolecular hydrogen bond. As a result, the molecule can exhibit ketoenamine–enolimine (or zwitterionic) tautomeric structures, as displayed in Scheme 2. Kamaal et al.^44^ reported the solvated form of the SB to exist in the zwitterionic form in the solid state, while Tahir et al.^45^ reported that the structure adopts the ketoenamine tautomer. The equilibria is significantly affected by interactions with solvent molecules, with proton-donating solvents tending to shift the equilibria toward the NH forms that were reported by the two sets of authors (zwitterion or ketoenamine).^46^ The tautomer adopted by the desolvate of the SB has yet to be reported but has the potential to shift to the enolimine form.

**Scheme 2:**
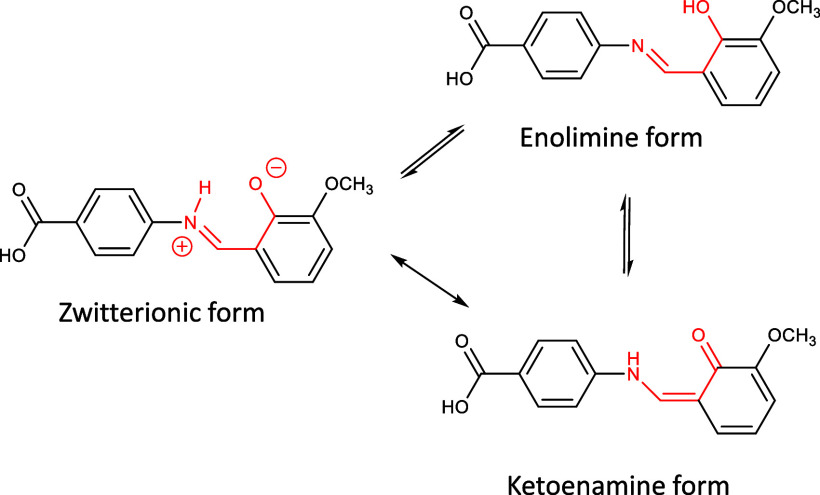
Ketoenamine–Enolimine Tautomerism of the Reported Schiff Base

On first inspection, the SB satisfied our requirements for a cheap and easy-to-manufacture model compound with the chemical complexity found in typical small active pharmaceutical ingredients. Kamaal et al.^44^ synthesized and isolated the SB in a small-scale reactive crystallization (15 mL), in which *o*-vanillin was added to a hot-stirred solution of PABA in methanol (0.5 mol/L), followed by heating under reflux for 90 min (with a precipitate forming after 1 h). The mixture was then cooled to room temperature, the mother liquor filtered off, and the crystals washed with methanol. Tahir et al.^45^ also synthesized the compound using a similar procedure but at a 14-fold reduction in reagent concentrations and double the reflux duration. Methanol was removed under reduced pressure before an aqueous workup was performed. Additional specifics were not given for either method, i.e., seeding/mixing/addition/cooling regimes and vessel type/geometry.

Our initial attempts to isolate the SB according to the more concentrated literature method of Kamaal et al.^44^ were unsuccessful in that the crystallization set solid. The alternative method of Tahir et al.^45^ is too dilute, and any efforts to concentrate the mixture by evaporation would be challenging at scale. Therefore, in the search for a suitable model solvate compound, we inadvertently encountered an interesting process development case. Reactive crystallizations tend to be challenging to scale up due to the number of simultaneous rate processes involved (reaction, crystallization, mixing). This paper describes the successful process development of a reactive crystallization protocol for the SB at a 1 L scale and its kinetic and structural characterization relevant to being a potential drying model compound.

## Materials and Methods

### Reactive Crystallization Apparatus

The apparatus (Figure 1) consisted of a fully automated 1 L jacketed HEL (Hazard Evaluation Laboratory Ltd.) calorimeter (model E227B Simular) with temperature control via an in situ PT100 temperature probe, Julabo FP32 heater/chiller, and PC installed with HEL WinISO (version 2.2.30.3) process control software. The vessel was equipped with a 40 mm diameter four-blade 45° pitched impeller made from polytetrafluoroethylene (PTFE), operated at the maximum speed of 600 rpm, and a vent condenser kept at 8 °C by a second heater/chiller (Huber Ministat) to condense evolved vapors. Any reagent addition to begin with was manual, but as development progressed, this was regulated by using a Knauer AZURA P4.1S pump with a 10 mL/min pump head. The apparatus was contained within a bunded walk-in fume hood. The vessel headspace was maintained under a nitrogen blanket.

**Figure 1:**
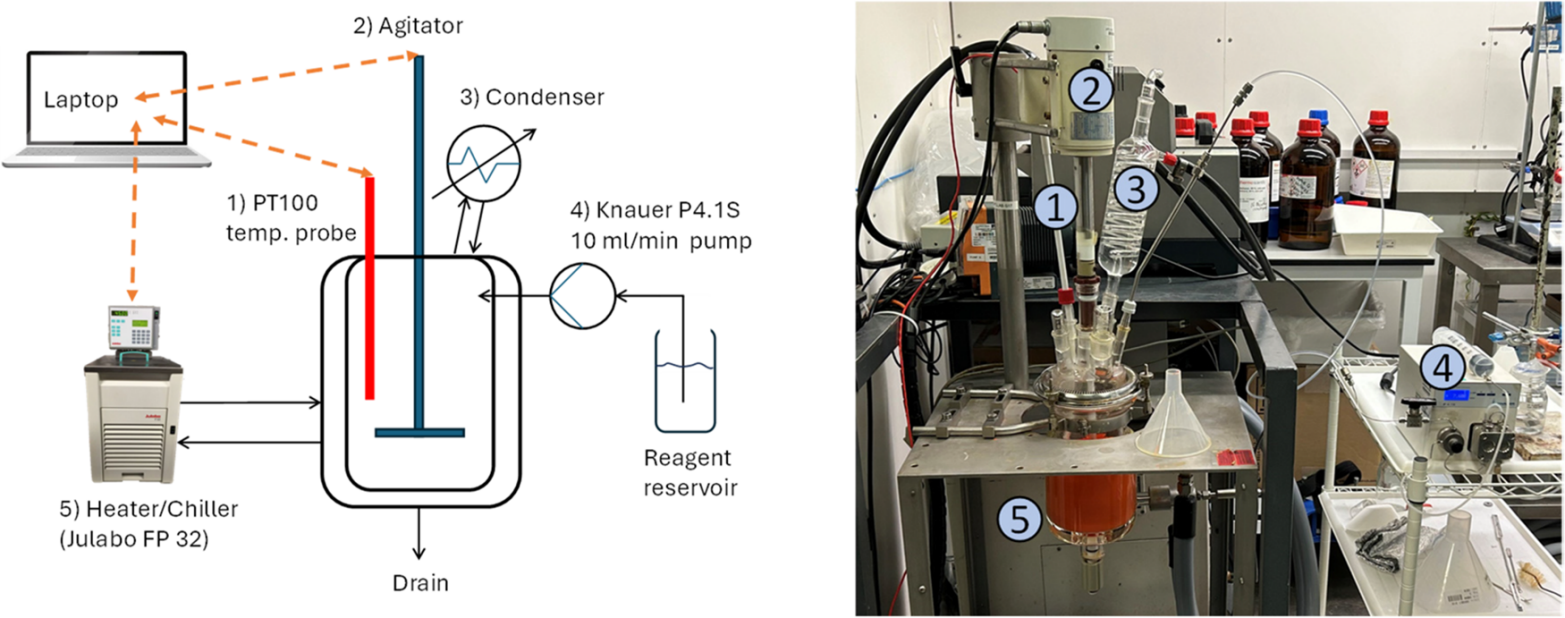
Fully automated 1 L jacketed HEL calorimeter inside a bunded walk-in fume hood.

### Reactive Crystallization Process

To produce 120 mg/mL of SB in 700 mL of methanol, *o*-vanillin (44.4 g, 0.292 mol, 99%, Fluorochem Ltd.) was dissolved in 350 mL of methanol (99% HPLC grade, Sigma-Aldrich) and heated to 60 °C. The mixture was seeded with approximately 10 g of the dry SB solvate (roughly 5 g dissolves at 60 °C; undissolved portion equates to 6 wt % of the theoretical maximum product mass of 86.8 g, accounting for that retained in solution). PABA (44 g, 0.292 mol, 99%, Fluorochem Ltd.) was dissolved in 350 mL of methanol and charged into the vessel using a slow profiled addition. Initially, aliquots were charged every 3 min, starting with 20 × 5 mL, then 10 × 10 mL, and the remaining fluid in 20 mL aliquots. Later in development, the PABA solution was pumped in using the Knauer pump over ∼2.5 h according to the addition profile in Figure 2. On completion of the addition, the orange slurry was held at 60 °C for 30 min and then cooled at 0.1 °C/min to room temperature. The crystals were vacuum-filtered in a Buchner funnel using Whatman grade 1 filter paper, followed by washing in pure methanol to preferentially remove PABA and *o*-vanillin, which are an order of magnitude more soluble than the SB. The product was then slurried with roughly 250 mL of methanol to prevent desolvation and transferred to a 1 L flask. In total, using the revised process route, four batches of material were produced. All materials were used as supplied.

**Figure 2:**
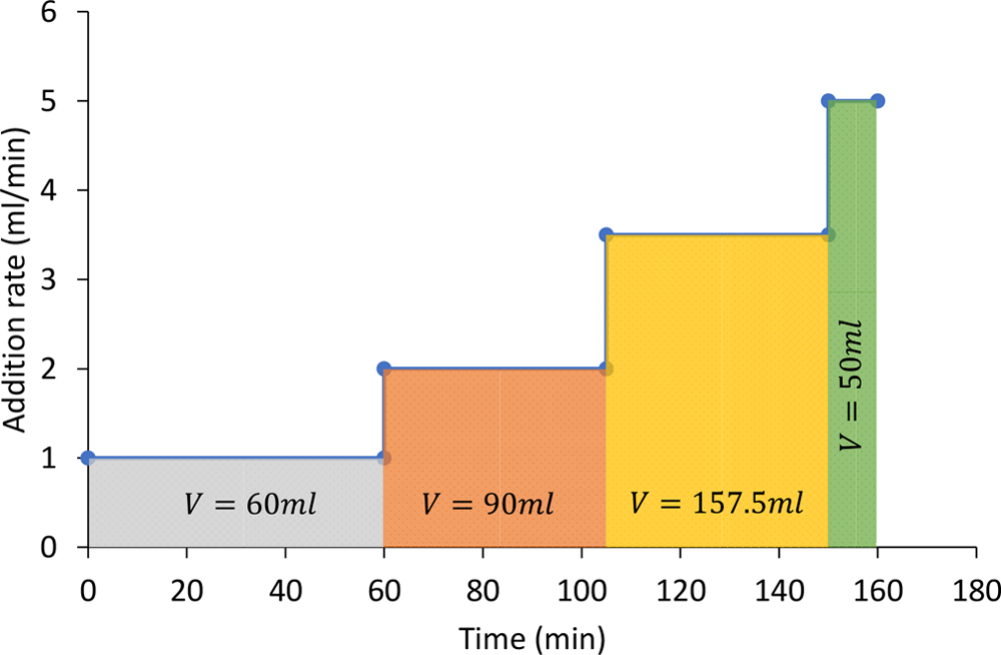
Addition profile of 44 g of PABA in 350 mL of methanol over 2.5 h into 44.4 g of *o*-vanillin in 350 mL of methanol, seeded at 6 wt % expected product yield.

### Product Yield

For the final batch of material, the yield of the product was determined. The washed slurry was filtered and dried in a Powder Systems Limited (PSL) GFD Lab 010 series agitated Nutsche filter-dryer. The slurry was filtered under a nitrogen pressure of 0.2 bar (above cake) and subsequently dried under an 8 L/min nitrogen flow (through-flow from above) with the outlet maintained at atmospheric pressure. No agitation was used. To prevent desolvation of the solvate, the temperature of the nitrogen flow and jacket of the vessel were maintained at 25 °C. The progression of drying was monitored using an in-bed thermocouple positioned 5 mm above the filter plate. Drying was stopped when the temperature of the cake stabilized at that of the incoming nitrogen. The mass of the material recovered was noted. To accurately determine the yield, the solvent content of the cake was measured. Three 1.5 g core samples were dried at 40 °C in an Ohaus MB90 moisture analyzer to remove any residual liquid methanol droplets (without inducing desolvation). The solvent content was averaged over three experiments. From this, the mass of “dry solvate” and yield was calculated.

### Product Purity

A ^1^H NMR spectrum was recorded on a Bruker 400 MHz spectrometer for the final batch of material. Roughly 50 mg of SB was dissolved in 0.6 mL of DMSO-*d*_6_. Spectral data were reported in ppm relative to the solvent.

### Solubility Measurements

Polythermal crystallization experiments were performed using a Technobis Crystal 16.^47^ The unit enables 16 1.5 mL HPLC vials, separated into four blocks, to undergo controlled heating and cooling histories while being magnetically stirred. Crystallization was monitored using a turbidimetric system.

Solutions of SB solvate in methanol were prepared at concentrations between 2 and 14 mg/mL on a 20 mL scale. A quantity of the SB solvate was dried on a filter under room temperature nitrogen to remove surface methanol. The dried SB was weighed into vials using a 0.1 mg accurate balance, followed by the addition of the solvent by mass. The vials were capped and heated to 60 °C on a stirrer hot plate, agitating at 400 rpm until all material dissolved. The solutions were transferred to 1.5 mL vials using a Fisher brand 100–1000 μL micropipette with preheated pipet tips (60 °C) to avoid unwanted crystallization.

The vials were first heated to 63 °C and maintained here for 1 h to ensure complete dissolution, followed by two cooling/heating cycles over the range 5–63 °C at 0.25 °C/min. Solutions were held for 30 min at the temperature limits. Stirring (via micro magnetic stirrer bars) was maintained at 700 rpm throughout. The dissolution point was detected from the change in turbidity measured by the device (when the light transmission was 100%).

### Thermal Analyses

Nonisothermal thermogravimetric analysis (TGA) and differential scanning calorimetry (DSC) were performed simultaneously using a Mettler Toledo TGA/DSC 1. Approximately 10 mg of surface-dried SB solvate was loaded into sealed 100 μL aluminum crucibles and subsequently exposed to a heating rate of 10 °C/min from 30 to 150 °C. Nitrogen was used as the purge gas.

### Optical Microscopy

Images of the crystals produced in the initial experiment were taken on a ZEISS Axiolab 5 light microscope fitted with a ZEISS Axiocam 512 color camera.

### Scanning Electron Microscopy (SEM)

SEM images of the product crystals produced from the revised process were taken on a Hitachi TM3030 tabletop microscope with a beam voltage of 15 kV. Samples of SB solvate were surface-dried of methanol prior to analysis.

### X-ray Diffraction (XRD)

The identity of the material was confirmed by comparing the material’s XRD pattern to the references from the CCDC database (Refcode: HISDOF and MONPEO). All samples used in the XRD work were methanol-wet, as they were transferred directly from stored SB solvate slurries. Powder patterns were measured at 25 °C using a 3 min scan on a Bruker D8 ADVANCE diffractometer with a LynxEye detector and nickel filter, λ­(Cu Kα) = 1.5418 Å, θ/2θ scan from 5° to 50°, step size 0.037° 2θ.

Nonisothermal X-ray diffraction measurements were conducted in situ on an Empyrean diffractometer (Malvern PANalytical, Malvern, UK). Starting with a methanol-wet sample, a 3 min scan was taken at 25 °C over 5–50° 2θ with a step size of 0.026° 2θ. The temperature was then increased in 10 °C increments up to 95 °C at a ramp rate of 5 °C/min with a 3 min scan taken at each increment.

The desolvation rate of the SB solvate as a function of temperature was investigated using an Ohaus MB90 moisture analyzer. Approximately 2 g of surface-dried solvate was charged in an aluminum sample tray and dried at 80 °C. Samples were taken every 10 min over 80 min by opening the dryer, mixing/shaking the material on the sample pan, then sampling (to ensure uniformity). Powder XRD patterns of the samples were taken ex situ using the same Bruker D8 instrument with the same method.

## Results and Discussion

Our initial experiment was conducted in line with the method described by Kamaal et al.,^44^ at the same reagent concentrations (0.5 mol/L) but at a 30× scale. Where experimental detail was absent (agitation, seeding, reagent addition protocol, vessel geometry), assumptions were made, and typical batch processing principles were followed. During the experiment, the addition of solid *o*-vanillin (over approximately 20 s) into an unseeded solution (due to no prior product material) of PABA in methanol (0.5 mol/L), stirred at 300 rpm and held at 60 °C, resulted in the product crashing out of solution and setting solid even before addition of *o*-vanillin had finished. The flow at the wall of the vessel and the surface of the slurry had ceased completely (Figure 3), indicating the fluid had developed a high yield stress and formed a cavern system (stagnant fluid with a mobile zone around the agitator).^48^ Attempts to remobilize the stationary fluid at the maximum agitation rate of 600 rpm were unsuccessful. The agitator was turning with a cylindrical “lollipop” caked around the impeller inside the “cavern”.

**Figure 3:**
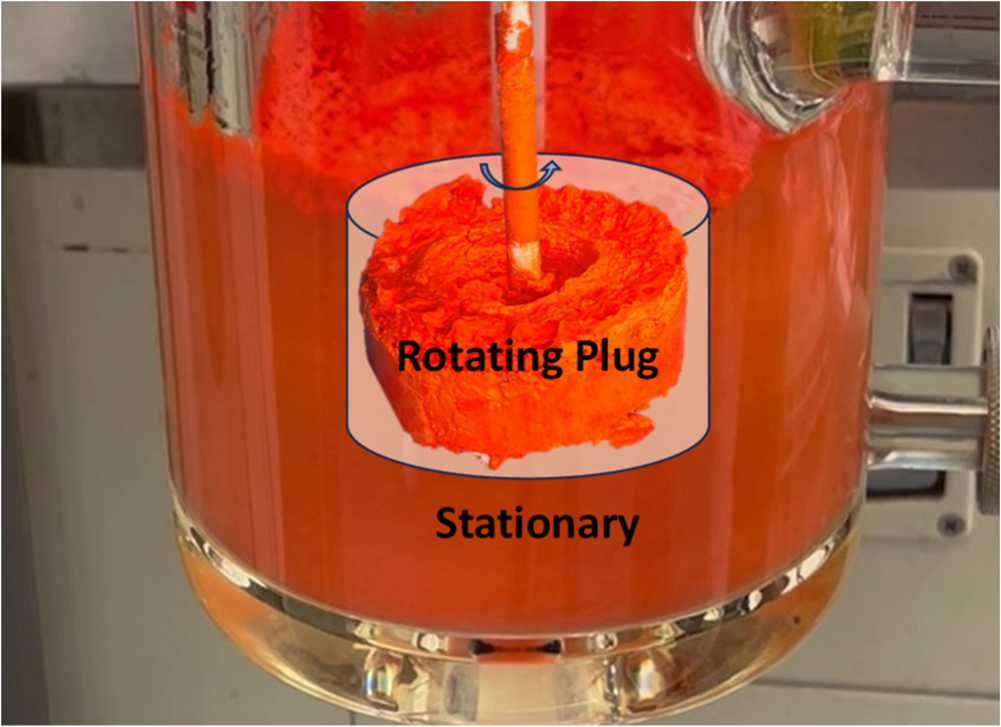
First attempt at reactive crystallization to form the SB solvate at 0.5 mol/L.^44^ The product “set-solid” after rapid addition of solid *o*-vanillin to a solution of PABA in MeOH. Material at the vessel wall remained stationary, while the agitator continued rotating along with a plug of solid attached to it (“lollipop”).

Also, the crystals obtained were bright orange. These outcomes were unexpected and did not reproduce the experimental results obtained by Kamaal et al.^44^ who found “colorless prismatic crystals after slow evaporative cooling”. Tahir et al.^45^ found “light yellow needles” from their dilute process. The synthesis of a similar Schiff-base by Plyuta et al.^46^ from *o*-vanillin and 2-aminobenzylalcohol (vs 4-aminobenzoic acid in this study) in methanol, yielded orange needle crystals, similarly to our work, which is typical of Schiff bases.

Optical microscopy images from the first attempt (Figure 4) revealed the product to be very fine needles (∼50 μm length); i.e., the crystals have a high aspect ratio (*L*/*W* ≈ 8). Muller^49^ investigated the rheology of batch crystallizations for caffeine, a model system which also forms high-aspect-ratio needles, and observed that crystals with this morphology can form “structure” that possesses a yield stress. The assumption was that at high supersaturation, “spotwelds” form on the contact points between nucleated crystals and that the size and strength of these spotwelds increase with supersaturation.

**Figure 4:**
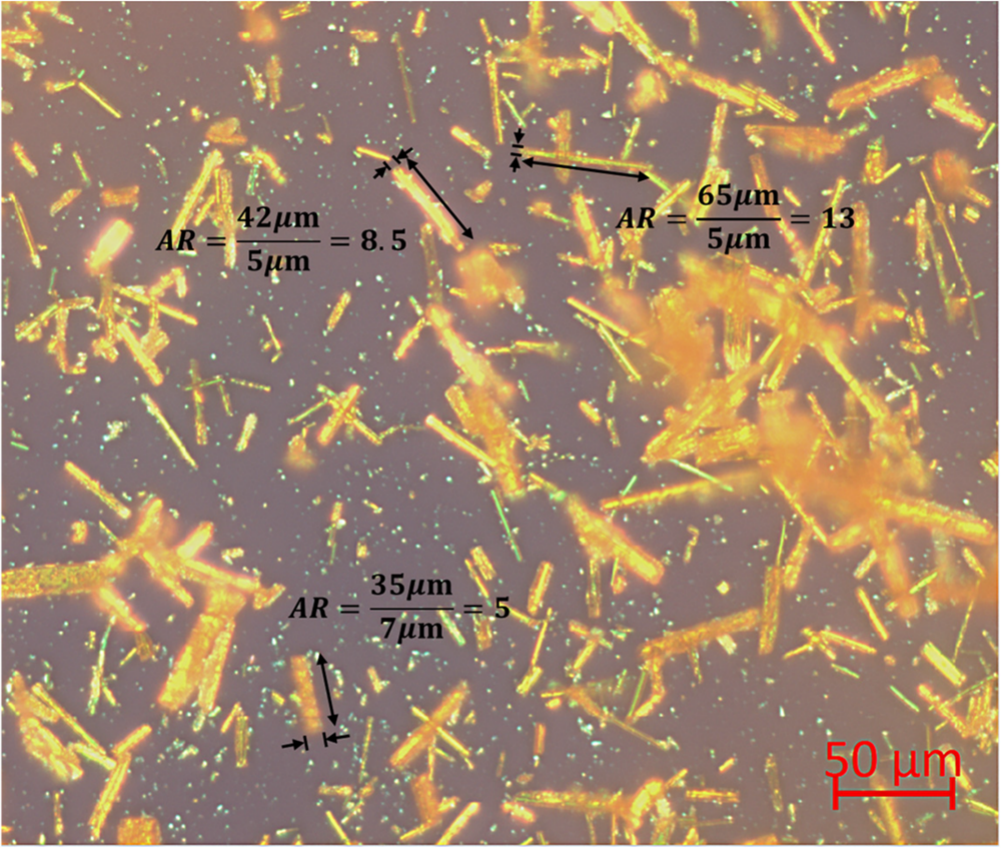
Optical microscopy images of the SB crystals from the initial experiment showing their high-aspect-ratio morphology, *L*/*W* ≈ 8.

In our initial experiment, the direct scale-up of the process from Kamaal et al.^44^ to a 0.5 L scale led to an extremely high supersaturation, resulting from the lumped addition of *o*-vanillin into PABA. The product concentration was 150 mg/mL (assuming all reagents consumed). This compares to a solubility of only 14 mg/mL in methanol at 60 °C, indicating a roughly 10-fold supersaturation! The water produced as a byproduct of the reaction is unlikely to affect the reaction mixture solubility as only 7 μL/(mL MeOH) is generated at 100% conversion. In the synthesis by Tahir et al.,^45^ the maximum product concentration is in the order of our measured solubility limit at 60 °C, indicating a more controllable level of supersaturation. Our solubility measurements are depicted in Figure 5. The data was fitted to the van ‘t Hoff equation defined by eq 1, which is a suitable fit to the data over the temperature range investigated, giving an enthalpy of crystallization of $\Delta H_{L\to S}=-35.9\;kJ/mol$ .

1 $$\ln\left[\frac{x\left(T\right)}{x\left(T_{ref}\right)}\right]=-\frac{\Delta H_{L\to S}}{R}\left(\frac{1}{T}-\frac{1}{T_{ref}}\right)$$

The solid state of the SB in the resulting mixture was not established, as the product was difficult to process due to its gelatinous structure. A sample of the solid was nevertheless used as seed material in our next crystallization attempt, which produced the desired solvate phase.

**Figure 5:**
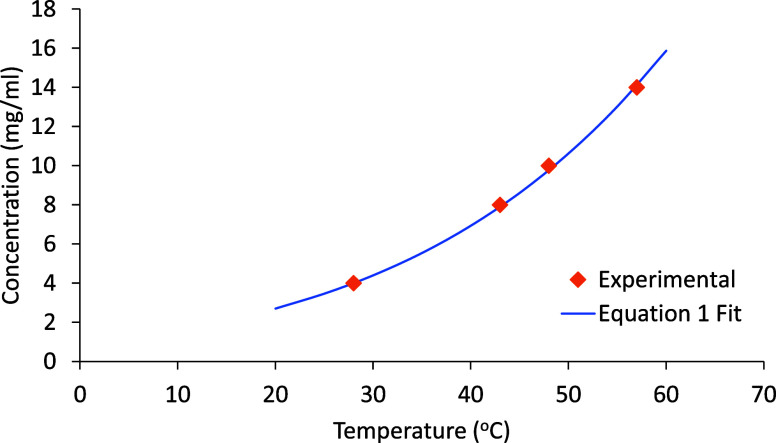
Solubility of the SB solvate in methanol. Data fitted to a van ‘t Hoff thermodynamic model (eq 1), giving an estimate for $\Delta H_{L\to S}=-35.9\;kJ/mol$ .

The process scheme (Figure 6) shows the mechanisms that conspired against the initial process; *o*-vanillin was rapidly added to a solution of PABA. PABA is an acid and as such catalyzes the reaction.^50^ So, starting with a high concentration of PABA results in maximum reaction rate. This was exacerbated by the speed of addition, which provided high concentrations of both reagents. The resulting high supersaturation then drove first nucleation and second rapid growth of high-aspect-ratio particles. Under such conditions, the high-aspect-ratio crystals “spotweld”. Muller^49^ showed that for high-aspect-ratio crystals, the propensity of a system to form “structure” is related to the Nienow number *Ni*

2 $$Ni=\frac{\rho N^{2}D^{2}}{X^{2/3}}$$

where ρ is the fluid density, *N* is the agitation rate, *D* is the agitator diameter, and *X* is the solute loading. The group expresses the ratio of turbulent forces acting on a structure to its cohesive force (yield stress), and Muller^49^ demonstrated the yield stress to be proportional to the solute loading. Crystallizations of high-aspect-ratio crystals operated at a *Ni* > 30 are expected to break up any formed structure and remain mobile throughout the process. In our case, *Ni* = 1.1 (ρ = 792 kg/m^3^, *N* = 5 rev/s (300 rpm), *D* = 0.04 m, *X* = 150 mg/mL), indicating that structures up to the size of the vessel are stable and cannot be destroyed (hence, the high degree of immobility in our initial experiment). Even the solid around the agitator could resist the agitator movement, and a cylindrical plug, a “lollipop”, rotated within the otherwise stationary bulk.

**Figure 6:**
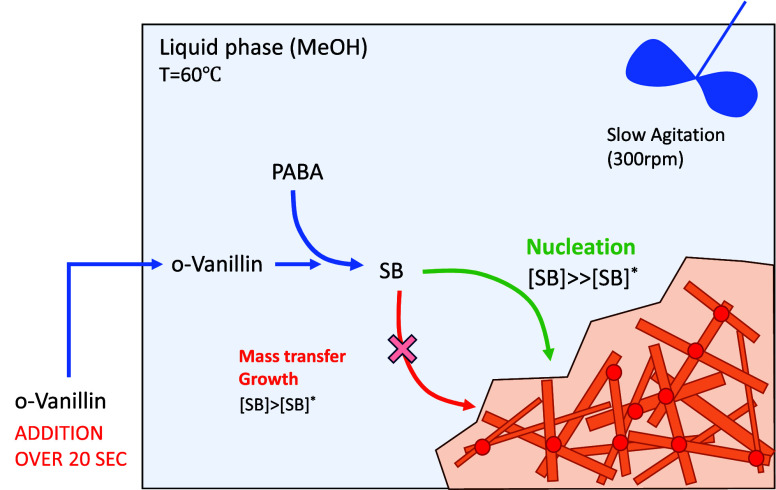
Process scheme of the initial attempt to produce the Schiff-base solvate; *o*-vanillin was added rapidly to a solution of PABA in methanol (* represents equilibrium). Red dots at crystal junctions represent “spotwelding”.

### Process Development

Several alterations to the process were introduced to avoid structure formation (Figure 7). First, reagent addition was reversed with PABA fed into *o*-vanillin. Second, the vessel was operated as a fed-batch system with PABA slowly dosed at a controlled rate (in solution). Kinetically, the reaction is fast, with both PABA and the SB catalyzing the imination reaction as acids.^50^ In contrast, the crystal growth kinetics are slow, so controlling the concentration of PABA in solution by implementing the above changes reduces the rate of generation of SB (to match growth rate) and in turn the degree of supersaturation in the vessel.

**Figure 7:**
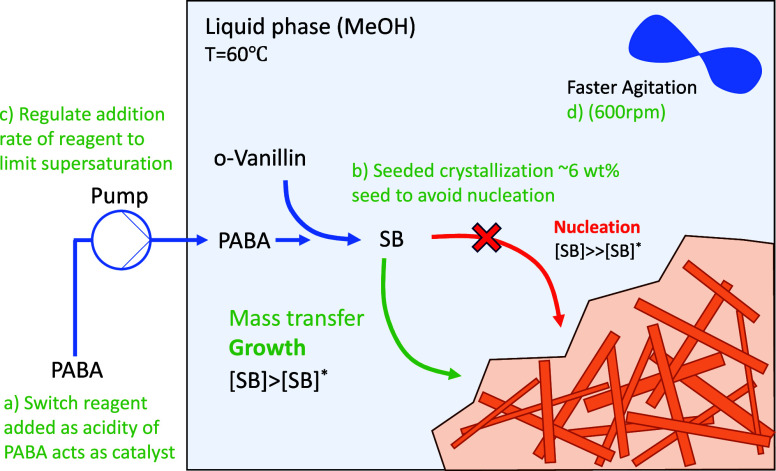
Representation of the process development to avoid the formation of the structure by (a) switching reagent addition (PABA into *o*-vanillin); (b) seeding at 6 wt % expected yield; (c) controlling the addition rate of PABA; (d) increasing the agitation rate to 600 rpm (* represents equilibrium).

Another change was to seed the crystallization (6 wt % of the maximum product mass) at 60 °C prior to PABA addition. By both regulating the level of supersaturation and seeding the crystallization, excessive primary nucleation, which generates fine crystals prone to spotwelding, is suppressed, and crystal growth becomes the dominant mechanism for removal of solute from the solution. The relatively high seed loading, which is not uncommon for reactive crystallizations (5–10%),^51,52^ enables a faster rate of crystallization, thus allowing a faster rate of addition.^53^ This is vital when PABA is first charged due to the reaction rate being at its maximum (high excess of *o*-vanillin). The quantity of seed actually needed is a balance between the PABA addition rate and the rate of crystallization, with the latter governed by the surface area of seed crystals (PSD).

Furthermore, the addition profile of PABA is ramped, as depicted in Figure 2. Initially, the available seed surface to deposit new material on is at its minimum, so the addition rate must be at its slowest to match the limiting crystallization rate, thus preventing high supersaturation. Over time, the addition rate can be increased without generating excessive supersaturation, as the available seed surface to deposit the solute increases.

An additional benefit of the reduced concentration of PABA and therefore SB in solution is that potential impurity formation rates are lower, e.g., a reduced rate of esterification of both acid components with methanol, assuming the rate depends on the concentration of [*H* ^+^] as was the case with maleic acid observed by Ashworth et al.^54^

Finally, the agitation rate was doubled to 600 rpm to break up any structure as it is formed. For the revised process, the Nienow number was *Ni* = 16 (ρ = 792 kg/m^3^, *N* = 10 rev/s (600 rpm), *D* = 0.04 m, *X* = 28 mg/mL). A maximum supersaturation ratio *C*/C* of 2 was assumed. Under the new operating conditions (*Ni* = 16), the risk of structure formation persists, and indeed, we observe that the slurry remains soft and sticky, especially at the start of PABA addition. Complete mobility is generally achieved only when *Ni* > 30. Eventually, the slurry thins out, and crystals can settle out after agitation is stopped and can be resuspended with ease. Mixing could be further improved by introducing baffles to increase turbulent mixing. The PT100 temperature probe fulfills this function to a degree.

SEM images of the crystals produced using the revised process are displayed in Figure 8. Compared to the first experiment (Figure 4), the crystals have a higher aspect ratio due to their increased length (8 vs 20), with this attributed to the improved control of product saturation, preventing primary/secondary nucleation and enabling crystal growth of the added seeds.

**Figure 8:**
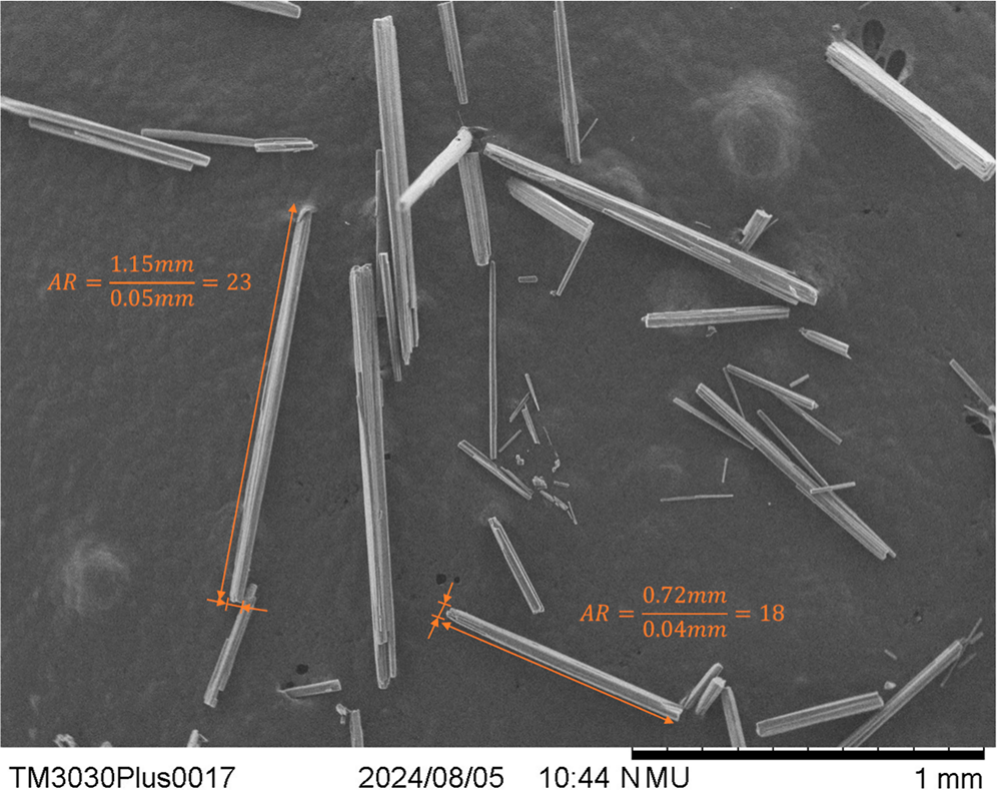
SEM images of the SB crystals produced using the revised process, showing their high-aspect-ratio morphology *L*/*W* ≈ 20.

The yield of the revised process was 93.4% (max theoretical yield is 97.5%—2.5 mg/mL of product retained in solution), as measured by the mass of “dry solvate” obtained in the final batch of material. This represents an improvement over the small-scale literature synthesis by Kamaal et al.^44^ and Tahir et al.^45^ (78% and 66%, respectively).

The product identity was confirmed using powder X-ray diffraction. As shown in Figure S1, there is a good match for peaks below 25° 2θ compared to the experimentally determined single-crystal patterns from Kamaal et al.^44^ and Tahir et al.^45^

A qualitative assessment of product purity was undertaken using ^1^H NMR. The spectrum of the SB product (Figure S2) displays all expected peaks (shifts labeled on the SB structure in Figure S2)—^1^H NMR (400 MHz, DMSO-*d*_6_): δ 12.85 (s, 1H), 9 (s, 1H), 8.05 (s, 2H), 7.5 (s, 2H), 7.3–7.2 (m, 2H), 6.95 (s, 1H), 3.8 (s, 3H). Minor residual PABA and *o*-vanillin impurity peaks as well as solvent peaks (MeOH and DMSO) have been identified. Despite the risk of esterifying the carboxylic acid moieties on PABA and the SB with methanol (due to elevated temperatures associated with reflux), there is no sign of these potential impurities.

TGA (Figure S3) yielded a mass loss of methanol between 95 and 120 °C corresponding to 0.117 kg/kg desolvated SB, confirming a 1:1 molar ratio of SB to methanol (0.118 kg/kg theoretical). Despite the fact that a quantitative assessment of product purity was not conducted, the mass loss by TGA is within 1% of the stoichiometric value. Both PABA and *o*-vanillin do not form solvates with methanol, so if there was a significant unreacted portion of either reagent, this would be highlighted by a reduction in the normalized solvent loss (kg/kg desolvated SB).

DSC data displays a single endothermic desolvation peak at ∼110 °C with a corresponding heat of desolvation of 45.6 kJ/mol MeOH. When compared to the heat of evaporation of pure methanol at 25 °C (37.34 kJ/mol^55^), it is evident that methanol is more strongly bound as a solvate.

### In Situ XRD Drying Study

To evaluate the desolvation behavior as a function of temperature, powder XRD patterns were recorded on a hot stage for temperatures between 25 and 100 °C (Figure S4). Over the time scale of the XRD measurements, no desolvation was observed below 75 °C, while partial desolvation occurred at 85 °C with the growth of peaks at 15° and 25.5°. Complete desolvation was achieved at 95 °C with the disappearance of two prominent peaks at 10° and 17.5°. The XRD analysis clearly identified the desolvated form, distinguished by two prominent peaks at 15° and 25.5°, along with the complete disappearance of peaks associated with the solvated material. Notably, this desolvated form has not been previously reported. The desolvation mechanism according to the well-cited “Rouen 96 model”^56^ classification is likely of the destructive–reconstructive type due to the rapid pattern changes between 85 and 95 °C. In this case, the supramolecular packing collapses as the solvent is released, but the SB host quickly rearranges into a new structure without the formation of an amorphous intermediate.

### Dynamic Drying Behavior

The isothermal desolvation kinetics shown in Figure 9 link the structural changes seen in Figure S4 to the solvate composition by intermittent sampling during drying and recording XRD patterns. As shown in Figure 9, the drying rate is strongly temperature-dependent. At room temperature, desolvation takes 1–2 days. Between 70 and 90 °C, the desolvation time decreases significantly, from 4 h to just <1 h. The desolvated material also exhibits a distinctly lighter color (sample pans, Figure 9). The stability of the solvate out of solution under atmospheric conditions is desirable as it enables analytical characterization to be conducted without the structure drastically changing. Also, by altering the drying temperature, drying and desolvation behavior can be explored separately or coupled, enabling the material to be representative of different industrially relevant systems and their associated challenges: (i) low temperature (separate)—long desolvation times make determining end point difficult; ­(ii) high temperature (coupled)—controlling/preventing desolvation is challenging as particle beds are typically inhomogeneous.

**Figure 9:**
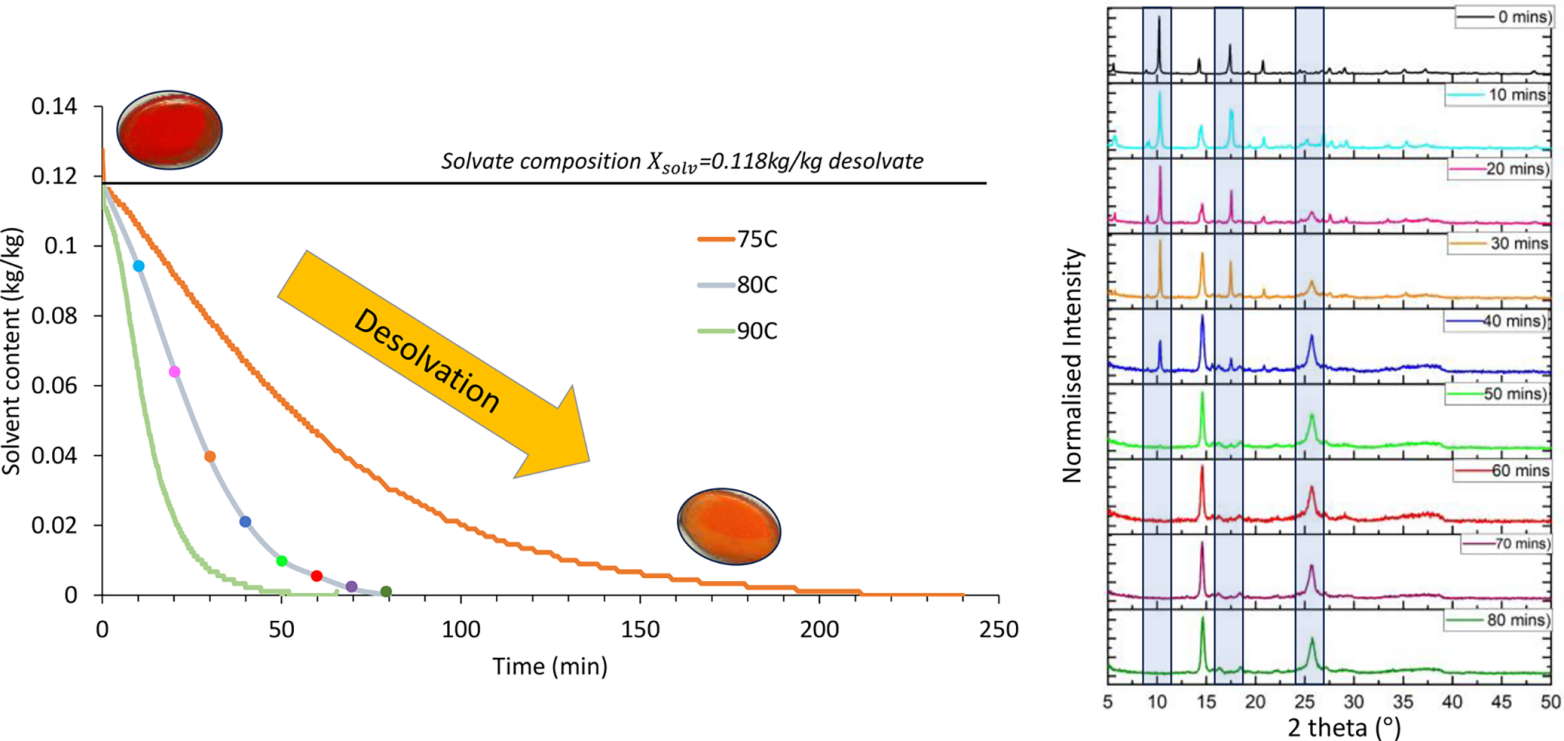
Schiff base desolvation at constant temperature. At 80 °C, combined analysis with XRD (ex situ) was undertaken. The different color dots along the drying curve (left) correspond to the colored XRD patterns (right). The three areas highlighted show the three peaks that indicate the extent of desolvation.

### Solvent Content Determination by XRD

Powder XRD spectra taken ex situ during drying (Figure 9) show a gradual transformation of the crystal structure from the solvate to a mixture and finally to a fully desolvated state. This transition is illustrated by the peaks at 10°, 17.5°, and 25.5°; the former two decrease in intensity as the solvent content reduces, and vice versa for the latter. At 10.1° and 25.5°, relative peak areas (to total area under patterns) were estimated manually, and Figure 10 shows a good correlation between the solvent content and peak area.

**Figure 10:**
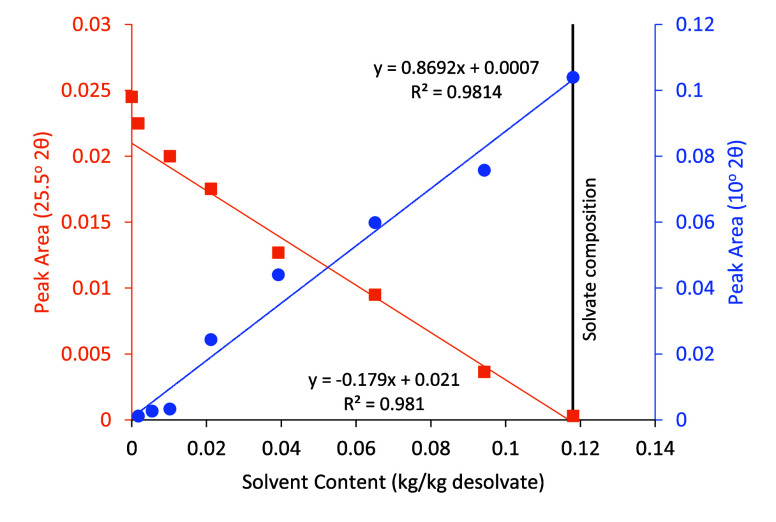
Powder XRD peak areas (relative to total area under the pattern) at 10° 2θ and 25.5° 2θ as a function of solvent content.

## Conclusions

We have developed a cost-effective and simple process for the manufacture of a model solvate compound for drying studies (4-[(3-methoxy-2-oxido-benzylidene)­azaniumyl] benzoic acid methanol monosolvate) that is representative of active pharmaceutical ingredients. The process required significant development in relation to regulating the generation and consumption of supersaturation to avoid rheological problems and ultimately resulted in a concentrated, high-yielding, and controllable process. The product yield was 93%, which represents an improvement of roughly 20% over previously reported small-scale experiments and highlights the successful scale-up of the process. Further work using inline PAT could be undertaken to optimize seed loading, PABA addition profiles, and mixing to enable a specific crystal size distribution to be targeted.

XRD analysis of the material confirmed that the target methanol solvate structure was obtained, and TGA confirmed the monosolvate stoichiometry expected. On desolvation, the crystal structure of the SB changes significantly, and we report the first XRD pattern for the “desolvated” Schiff base.

The Schiff base looks to be a promising candidate as a model solvate system for drying studies as (a) the material consists of high-aspect-ratio crystals typical of many organic products; (b) the solvate is stable at room temperature for significant periods of time, which facilitates both handling and analysis; (c) its desolvation temperature is above the boiling point of methanol, separating the drying of the free solvent from the period of desolvation; (d) the solvent content of the solvate can be assessed using loss on drying, and a method for determining solvent content from powder XRD spectra (via a calibration curve) is presented. Further work will be conducted to investigate the drying and desolvation behavior of this compound.

## Supplementary Material



## Notes

Data related to the analytical characterization of the Schiff base including x-ray diffraction patterns, ^1^H NMR spectra, and TGA/ DSC data (PDF)

## References

[1] Mann, J. Data Mining and Solid State Desolvation Pathways of Molecular Solvates. Ph.D. Thesis, Georgetown University, Washington D.C., 2023. https://repository.digital.georgetown.edu/handle/10822/1082510.

[2] Görbitz C. H., Hersleth H. P., Hersleth H. P. (2000). On the
Inclusion of Solvent Molecules
in the Crystal Structures of Organic Compounds. Acta Crystallogr., Sect. B: Struct. Sci..

[3] Byrn, S. ; Zografi, G. ; Chen, X. Solvates and Hydrates. In Solid State Properties of Pharmaceutical Materials; John Wiley & Sons, 2017; pp 38–47.

[4] Tieger E., Kiss V., Pokol G., Finta Z., Rohlíček J., Skořepová E., Dušek M. (2016). Rationalization
of the Formation and Stability of Bosutinib Solvated Forms. CrystEngComm.

[5] Brittain, H. G. ; Morris, K. R. ; Boerrigter, S. X. M. Structural Aspects of Solvatomorphic Systems. In Polymorphism in Pharmaceutical Solids; Brittan, H. G. , Ed.; CRC Press, 2009; pp 233–281.

[6] Stephenson G. A., Groleau E. G., Kleemann R. L., Xu W., Rigsbee D. R. (1998). Formation
of Isomorphic Desolvates: Creating a Molecular Vacuum. J. Pharm. Sci..

[7] Skořepová E., Václavík M., Jirát J., Poupon M., Ridvan L., Babor M., Šoóš M. (2022). Obeticholic
Acid Forms Two Series of Isostructural Non-Stoichiometric Channel
Solvates. Cryst. Growth Des..

[8] Han R., Qu H., Li Y., Liu Y., Tao T., Tang W., Li Z., Gong J. (2023). Development Strategies
of Polymorphs and Solvates for
Enhancing Powder Properties: A Case Study of Thiothiamine. Cryst. Growth Des..

[9] Black S. N., Wheatcroft H. P., Roberts R., Jones M. F., McFarlane I., Pettersen A. (2020). Cediranib Maleate – From Crystal Structure Toward
Materials Control. J. Pharm. Sci..

[10] MEKINIST (Trametinib) Label; Food and Drug Administration, revised April 2025. https://www.accessdata.fda.gov/drugsatfda_docs/label/2025/204114s038,217513s009lbl.pdf.

[11] Forxiga (Dapagliflozin) Public Assessment Report; European Medicines Agency, revised September 2012. https://www.ema.europa.eu/en/documents/assessment-report/forxiga-epar-public-assessment-report_en.pdf.

[12] JEVTANA (Cabazitaxel) Label; Food and Drug Administration, revised June 2023. https://www.accessdata.fda.gov/drugsatfda_docs/label/2023/201023s026lbl.pdf.

[13] PREZISTA (Darunavir) Label; Food and Drug Administration, revised March 2023. https://www.accessdata.fda.gov/drugsatfda_docs/label/2023/021976s06s068,202895s036lbl.pdf.

[14] DOXTERIC (Doxycycline Hyclate) Label; Food and Drug Administration. https://www.accessdata.fda.gov/drugsatfda_docs/label/2016/90431orig1s010lbl.pdf, revised April 2016.

[15] COUMADIN (Warfarin Sodium) Label; Food and Drug Administration, revised August 2017. https://www.accessdata.fda.gov/drugsatfda_docs/label/2017/009218s118lbl.pdf.

[16] CRIXIVAN (Indinavir Sulfate) Label; Food and Drug Administration. https://www.accessdata.fda.gov/drugsatfda_docs/label/2016/020685s078lbl.pdf, revised Sept 2016.

[17] Vosevi, Public Assessment Report; European Medicines Agency, revised June 2017. https://www.ema.europa.eu/en/documents/assessment-report/vosevi-epar-public-assessment-report_en.pdf.

[18] Wirth D. D., Stephenson G. A. (1997). Purification
of Dirithromycin. Impurity Reduction and
Polymorph Manipulation. Org. Process Res. Dev..

[19] Maini L., Braga D., Farinella F., Melotto E., Verzini M., Brescello R., Michieletto I., Munari I. (2018). Crystal Forms of Enzalutamide
and a Crystal Engineering Route to Drug Purification. Cryst. Growth Des..

[20] Sekiguchi K., Ito K., Owada E., Ueno K. (1964). Studies on the Method of Size Reduction
of Medicinal Compounds. II. Size Reduction of Griseofulvin by Solvation
and Desolvation Method using Chloroform (2). Chem. Pharm. Bull..

[21] Rocco W. L., Morphet C., Laughlin S. M. (1995). Solid-State Characterization of Zanoterone. Int. J. Pharm..

[22] Schmidt A. C., Niederwanger V., Griesser U. J. (2004). Solid-State Forms
of Prilocaine Hydrochloride. J. Therm. Anal.
Calorim..

[23] Braun D. E., Kahlenberg V., Gelbrich T., Ludescher J., Griesser U. J. (2008). Solid State Characterisation
of Four Solvates of R-Cinacalcet
Hydrochloride. CrystEngComm.

[24] Landgraf K. F., Olbrich A., Pauluhn S., Emig P., Kutscher B., Stange H. (1998). Polymorphism and Desolvation of Flupirtine Maleate. Eur. J. Pharm. Biopharm..

[25] Minkov V. S., Beloborodova A. A., Drebushchak V. A., Boldyreva E. V. (2014). Furosemide
Solvates: Can They Serve As Precursors to Different Polymorphs of
Furosemide?. Cryst. Growth Des..

[26] Suitchmezian V., Jeß I., Näther C. (2006). Investigations
on the Polymorphism
and Pseudopolymorphism of Triamcinolone Diacetate. Int. J. Pharm..

[27] Kemp I. C., Oakley D. E. (2002). Modelling of Particulate Drying in
Theory and Practice. Dry. Technol..

[28] Conder E. W., Cosbie A. S., Gaertner J., Hicks W., Huggins S., MacLeod C. S., Remy B., Yang B. S., Engstrom J. D., Lamberto D. J., Papageorgiou C. D. (2017). The Pharmaceutical
Drying Unit Operation:
An Industry Perspective on Advancing the Science and Development Approach
for Scale-Up and Technology Transfer. Org. Process
Res. Dev..

[29] Gaertner, J. ; Nere, N. K. ; Marek, J. C. ; Bordawekar, S. ; Mlinar, L. ; Diwan, M. ; Cao, L. Drying Case Studies. In Chemical Engineering in the Pharmaceutical Industry; am Ende, M. T. , am Ende, D. J. , Eds.; John Wiley & Sons, 2019; pp 847–860.

[30] Nere N. K., Allen K. C., Marek J. C., Bordawekar S. V. (2012). Drying
Process Optimization for an API Solvate Using Heat Transfer Model
of an Agitated Filter Dryer. J. Pharm. Sci..

[31] Lamberto D. J., Neuhaus J. (2021). Robust Process Scale-Up
Leveraging Design of Experiments
to Map Active Pharmaceutical Ingredient Humid Drying Parameter Space. Org. Process Res. Dev..

[32] Hsieh D. S., Gao Q., Huang M., DiMemmo L. M., Lindrud M., Razler T. (2017). From Drying
Kinetics, Solvate Structure, Particle Morphology, and Modeling to
Optimal Drying Protocol. Org. Process Res. Dev..

[33] Zhou G. X., Ge Z., Dorwart J., Izzo B., Kukura J., Bicker G., Wyvratt J. (2003). Determination
and Differentiation of Surface and Bound
Water in Drug Substances by near Infrared Spectroscopy. J. Pharm. Sci..

[34] Laurent S., Couture F., Roques M. (1999). Vacuum Drying of a Multicomponent
Pharmaceutical Product Having Different Pseudo-Polymorphic Forms. Chem. Eng. Process. Process Intensif..

[35] Lamberto D. J., Diaz-Santana A., Zhou G. (2017). Form Conversion and Solvent Entrapment
during API Drying. Org. Process Res. Dev..

[36] Adamson J., Faiber N., Gottlieb A., Hamsmith L., Hicks F., Mitchell C., Mittal B., Mukai K., Papageorgiou C. D. (2016). Development
of Suitable Plant-Scale Drying Conditions That Prevent API Agglomeration
and Dehydration. Org. Process Res. Dev..

[37] Airaksinen S., Karjalainen M., Räsänen E., Rantanen J., Yliruusi J. (2004). Comparison
of the Effects of Two Drying Methods on
Polymorphism of Theophylline. Int. J. Pharm..

[38] Touil A., Peczalski R., Zagrouba F. (2013). Monitoring of Theophylline Dehydration
in a Vacuum Contact Dryer by Near-Infrared Spectroscopy. Chem. Eng. Res. Des..

[39] Amira T., Roman P., Fethi Z. (2016). On-Line Monitoring
of Vacuum Drying
of Theophylline Using NIR Spectroscopy: Solid-State Transitions, Water
Content and Semi-Empirical Modeling. Drug Dev.
Ind. Pharm..

[40] Keavney, E. Approaches to Drying Hydrated Active Pharmaceutical Ingredients (APIs). Ph.D. Thesis, Trinity College Dublin, 2023. https://www.tara.tcd.ie/items/12821ba2-08cd-4a6a-897f-96b4a28aacce.

[41] Fonteyne M., Gildemyn D., Peeters E., Mortier S. T. F. C., Vercruysse J., Gernaey K. V., Vervaet C., Remon J. P., Nopens I., De Beer T. (2014). Moisture and Drug Solid-State
Monitoring
during a Continuous Drying Process Using Empirical and Mass Balance
Models. Eur. J. Pharm. Biopharm..

[42] Mann J. E., Gao R., London S. S., Swift J. A. (2023). Desolvation Processes in Channel
Solvates of Niclosamide. Mol. Pharm..

[43] Aitipamula S., Chow P. S., Tan R. B. H. (2014). Solvates
of the Antifungal Drug Griseofulvin:
Structural, Thermochemical and Conformational Analysis. Acta Crystallogr., Sect. B: Struct. Sci., Cryst. Eng. Mater..

[44] Kamaal S., Faizi M. S. H., Ali A., Ahmad M., Iskenderov T. (2018). Crystal Structure
of 4-[(3-Meth-Oxy-2-Oxidobenzyl-Idene)­Azaniumyl]-Benzoic Acid Methanol
Monosolvate. Acta Crystallogr., Sect. E: Crystallogr.
Commun..

[45] Tahir M. N., Ali A., Khalid M., Ashfaq M., Naveed M., Murtaza S., Shafiq I., Asghar M. A., Orfali R., Perveen S. (2023). Efficient
Synthesis of Imine-Carboxylic Acid Functionalized Compounds: Single
Crystal, Hirshfeld Surface and Quantum Chemical Exploration. Molecules.

[46] Plyuta N., Kokozay V., Cauchy T., Avarvari N., Goreshnik E., Petrusenko S. (2019). Solvent Dependent Prototropic Tautomerism
in a Schiff
Base Derived from o-Vanillin and 2-Aminobenzylalcohol. ChemistrySelect.

[47] Technobis . Crystal 16 Information. https://www.crystallizationsystems.com/products/crystal16/. Accessed on 20/03/2025.

[48] Nienow A.
W., Elson T. (1988). Aspects of
Mixing in Rheologically Complex Fluids. Chem.
Eng. Res. Des..

[49] Muller F. L. (2009). On the
Rheological Behaviour of Batch Crystallisations. Chem. Eng. Res. Des..

[50] Adesina, A. D. Synthesis of Schiff Bases by Non-Conventional Methods. In Schiff Base in Organic, Inorganic and Physical Chemistry; Akitsu, T. , Ed.; IntechOpen, 2022.

[51] Jiang M., Ni X. W. (2019). Reactive Crystallization of Paracetamol in a Continuous Oscillatory
Baffled Reactor. Org. Process Res. Dev..

[52] Wang H. Y., Ward J. D. (2015). Seeding and Optimization
of Batch Reactive Crystallization. Ind. Eng.
Chem. Res..

[53] Zhang F., Shan B., Wang Y., Zhu Z., Yu Z. Q., Ma C. Y. (2021). Progress and Opportunities for Utilizing Seeding Techniques in Crystallization
Processes. Org. Process Res. Dev..

[54] Ashworth I. W., Bush E., Chan L. C., Cherryman J., Cox B. G., Muir J., Korupoju S. R., Keshwan J. (2012). Where Has
My Acid Gone? Understanding the Self-Catalyzed Esterification of Maleic
Acid in Methanol During Salt Formation. Org.
Process Res. Dev..

[55] Felder, R. M. ; Rousseau, R. W. Elementary Principles of Chemical Processes, 3rd ed.; Wiley, 2005.

[56] Petit S., Coquerel G. (1996). Mechanism of Several Solid–Solid Transformations
between Dihydrated and Anhydrous Copper­(II) 8-Hydroxyquinolinates.
Proposition for a Unified Model for the Dehydration of Molecular Crystals. Chem. Mater..

