# Supplementary material for: Process Development of a Model Solvate for Drying Research
Source: Org Process Res Dev. 2025 Aug 26;29(9):2200–9. doi: 10.1021/acs.oprd.5c00095 (PMC12455650; doi:10.1021/acs.oprd.5c00095)
Supplement: Supplementary file 1 [file op5c00095_si_001.pdf]

Supporting Information

Process Development of a Model Solvate for Drying Research

*Nicholas H. McCarthy[1], Norah S. Alsaiari[1,2], Thomas Brown[3], Faiz M. Mahdi[1], Andrew E. Bayly[1], Sadie Finn[4], Frans L. Muller[1]**

[1]School of Chemical and Process Engineering, University of Leeds, Leeds, LS2 9JT, UK

[2]Department of Chemistry, College of Science, Princess Nourah bint Abdulrahman University, Riyadh 11671, Saudi Arabia

[3]Bragg Centre for Materials Research, University of Leeds, Leeds, LS2 9JT, UK

[4]Pharmaceutical Technology and Development, AstraZeneca, Macclesfield, SK11 2NA, UK

*Email: F.L.Muller@leeds.ac.uk

X-ray diffraction pattern of product compared to literature

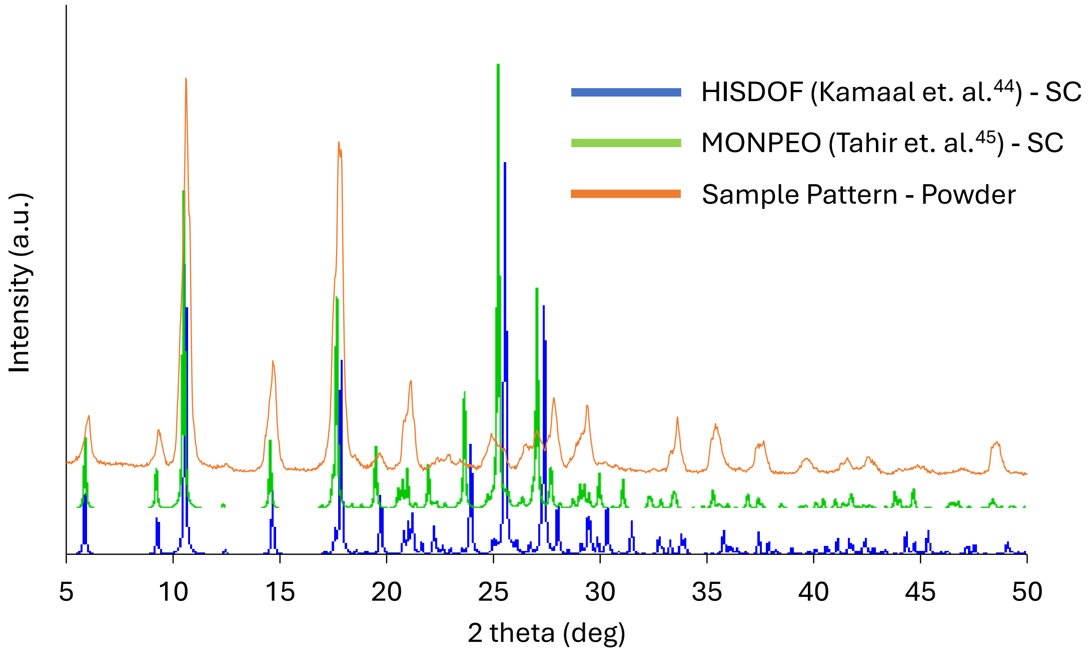

Figure S1. Powder x-ray diffraction pattern of the Schiff base solvate from this work compared to the single crystal (SC) patterns obtained by Kamaal[44] (CCDC: HISDOF) and Tahir et. al.[45] (CCDC: MONPEO).

^{1}H NMR of product

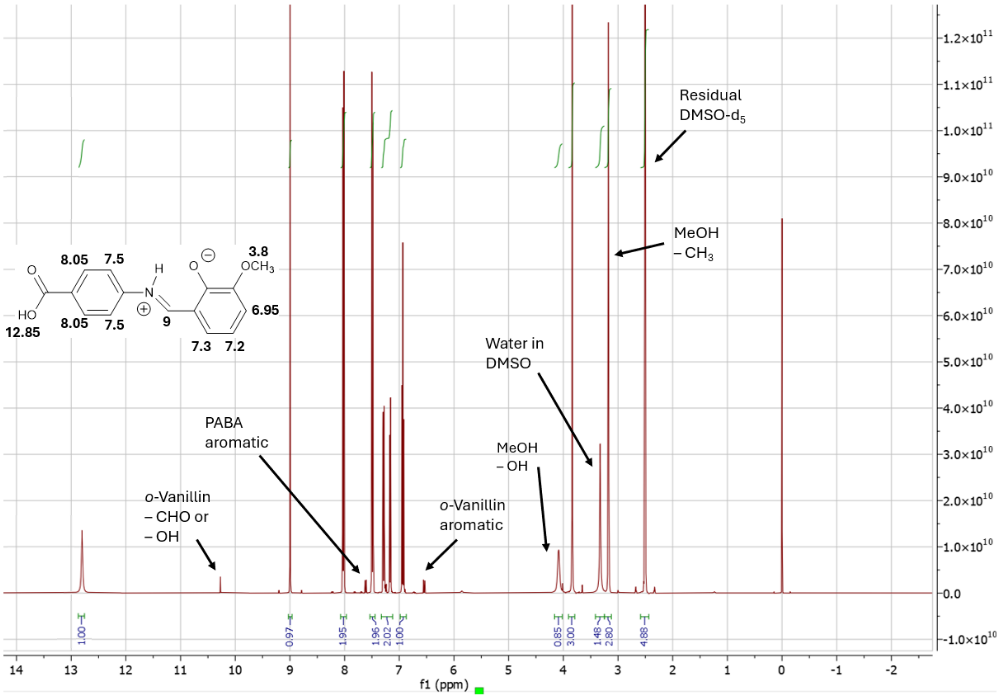

Figure S2. ^{1}H NMR of the Schiff base solvate. Peaks corresponding to the SB are labelled next to the specific protons on the molecular structure. Minor residual PABA and o-vanillin impurity peaks have been identified, as well as solvent peaks (MeOH and DMSO).

TGA/DSC of product

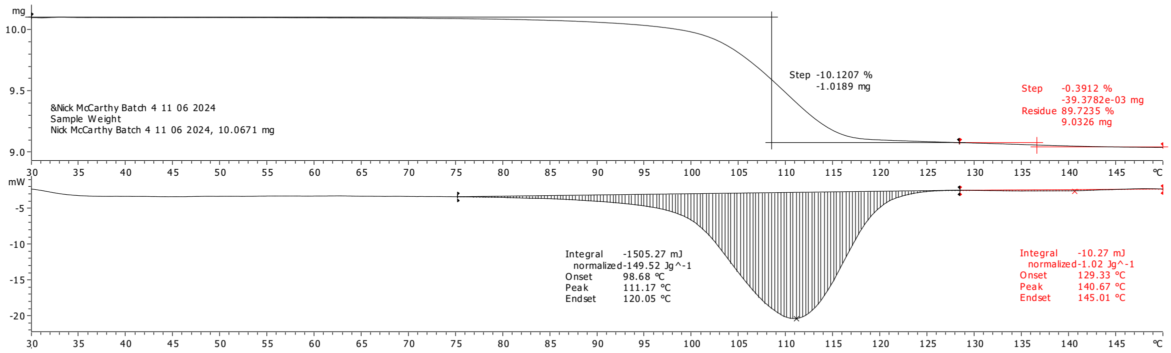

Figure S3. TGA (top) and DSC (bottom) of Schiff base solvate at 10℃/min heating rate.

Variable temperature powder x-ray diffraction of product

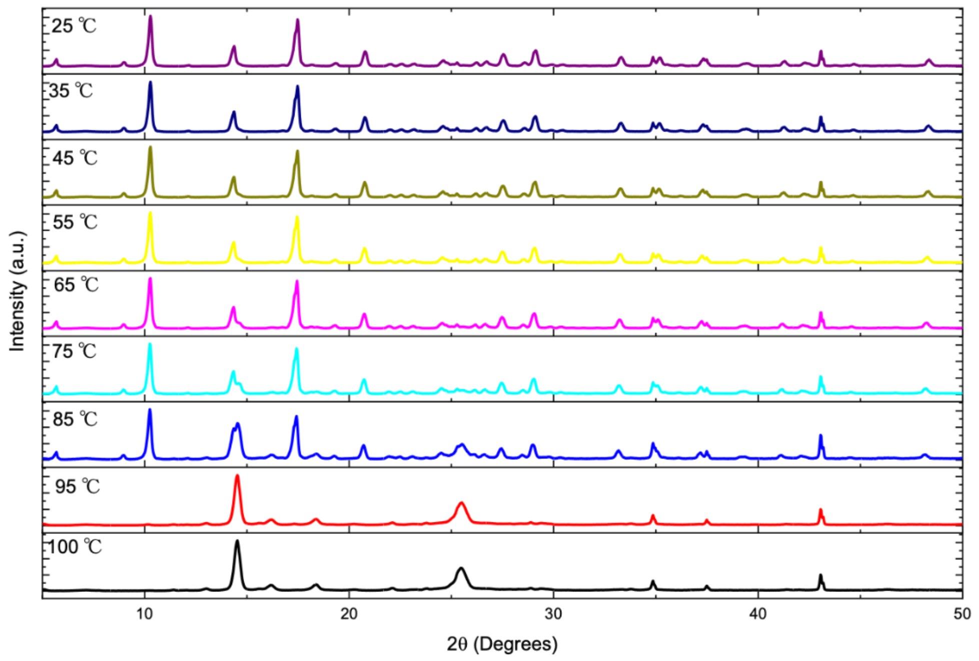

Figure S4. Powder XRD patterns of the Schiff base solvate recorded incrementally between 25 and 100 °C in situ.
